# R-Ras GTPases Signaling Role in Myelin Neurodegenerative Diseases

**DOI:** 10.3390/ijms21165911

**Published:** 2020-08-17

**Authors:** Berta Alcover-Sanchez, Gonzalo Garcia-Martin, Francisco Wandosell, Beatriz Cubelos

**Affiliations:** Departamento de Biología Molecular and Centro Biología Molecular “Severo Ochoa”, Universidad Autónoma de Madrid, 28049 Madrid, Spain; berta.alcover@cbm.csic.es (B.A.-S.); gonzalo.garciamartin@estudiante.uam.es (G.G.-M.); fwandosell@cbm.csic.es (F.W.)

**Keywords:** myelin, oligodendrocyte, neurodegeneration, PI3K/Akt/mTOR, ERK1/2-MAPK, Wnt/β-catenin, R-Ras

## Abstract

Myelination is required for fast and efficient synaptic transmission in vertebrates. In the central nervous system, oligodendrocytes are responsible for creating myelin sheaths that isolate and protect axons, even throughout adulthood. However, when myelin is lost, the failure of remyelination mechanisms can cause neurodegenerative myelin-associated pathologies. From oligodendrocyte progenitor cells to mature myelinating oligodendrocytes, myelination is a highly complex process that involves many elements of cellular signaling, yet many of the mechanisms that coordinate it, remain unknown. In this review, we will focus on the three major pathways involved in myelination (PI3K/Akt/mTOR, ERK1/2-MAPK, and Wnt/β-catenin) and recent advances describing the crosstalk elements which help to regulate them. In addition, we will review the tight relation between Ras GTPases and myelination processes and discuss its potential as novel elements of crosstalk between the pathways. A better understanding of the crosstalk elements orchestrating myelination mechanisms is essential to identify new potential targets to mitigate neurodegeneration.

## 1. Introduction

### 1.1. Myelination and Oligodendrocytes

Myelination is an essential process for the correct transmission of nerve impulses. Rapid and effective neural electric transmission through the axon is required for the correct integration of information. In the mammalian central nervous system (CNS), oligodendrocytes (OLs) are responsible for axon myelination in a complex process involving various cellular interactions [1]. OLs form myelin sheaths around axons to isolate them from extracellular space and provide them with metabolic support. Myelin sheaths are multilayered membranes that result from wrapping and compaction of the plasma membranes of OLs around axons [2,3]. Myelination facilitates the disposition of depolarization machinery in discontinuous zones between myelin sheaths known as nodes of Ranvier [4,5]. This organization guarantees that axonal membrane depolarization only occurs at discontinuous zones, resulting in a rapid and effective saltatory conduction of the nerve impulse through the axon, a feature believed to contribute to vertebrate evolution [6,7,8].

From a developmental point of view, OLs are generated from OL progenitor cells (OPCs) [9,10]. Before their differentiation, OPCs migrate along the CNS and begin to differentiate into mature OLs [11]. From the beginning of development, differentiation steps have been identified according to migratory capacity, morphologic complexity, gene expression, and expression pattern of specific markers [12,13,14,15]. The maturation of oligodendroglial cells involves a progressive modification of OL abilities. Their initial migratory and proliferation capacity is lost during maturation, as they progressively acquire an elaborate morphology [16,17].

In the myelination process, OLs are responsible for myelin development by extending processes that wrap around axons and form compact myelin sheaths. Myelination starts as OPCs extend and retract processes looking for axons that will be myelinated in the future [18,19]. Upon the first contact with the axolemma, the end of the OPC process retracts or stabilizes, forming a specialized membrane domain for continuous communication between the axon and the OL. Then, the future myelin sheath expands radially and longitudinally, simultaneously with the addition of a new membrane at the growth end of the internal tongue [20]. At the same time, the innermost layer of the sheath expands laterally, compacting between the anterior layer and the axon, wrapping around it. The addition of membrane at the expansion end requires transport of components that have been synthesized in the soma, through the oligodendroglial processes, which means an increase in energy expenditure for the OL. The cytoplasmic subdomains at the end of each myelin lamina remain in close contact with the axon, which is covered by them moving laterally and around it towards the future node of Ranvier [4], where they eventually form a series of closely juxtaposed paranodal loops. These multilayered membrane compartments (nodes) are distributed along the axon to provide metabolic support and protection from the extracellular space [21,22,23,24]. The importance of proper myelination is highlighted by the existence of many neurodegenerative diseases in which myelin deficits cause alterations in nerve impulse transmission.

### 1.2. Myelin Neurodegenerative Diseases

Myelin neurodegenerative diseases include a wide variety of disorders with different etiologies and manifestations, including sensory, motor, and cognitive alterations [25]. Some of the most important pathologies affecting the CNS are multiple sclerosis (MS), neuromyelitis optica (NMO), hypomyelinating leukodystrophies, and Charcot-Marie-Tooth (CMT) disease.

MS is the most prevalent neurodegenerative disease of the CNS in young adults, affecting more than 2 million people worldwide [26]. It is usually diagnosed around the age of 20–40 years, and it is 2–3 times more frequent in women than men [27]. Although the etiology remains unknown, it is believed to occur as a result of some combination of genetic and environmental factors [28]. Nowadays MS is characterized by demyelination with concomitant axonal and neuronal degeneration that causes a heterogeneous array of symptoms and signs [28,29,30]. NMO, previously called Devic’s disease, is considered to be a rare, autoimmune, demyelinating disease of the CNS which manifests as optic neuritis and acute transverse myelitis. This disease affects more than 2 million people worldwide and has a prevalence of 1–3 per 100,000, being more frequent in women than in men [31,32]. Another neurodegenerative disease is Pelizaeus–Merzbacher disease (PMD), a hypomyelinating leukodystrophy where the *PLP1* gene is mutated [33]. CMT disease is a phenotypically and genetically heterogeneous group of sensory and motor neuropathies [34].

## 2. Cell Signaling Pathways Involved in Myelination

From initial OPC proliferation to myelin sheath maintenance through adulthood, myelination requires a sophisticated interaction of multiple signaling pathways. The principal signaling pathways driving OL differentiation, myelination, and remyelination after injury are PI3K/Akt/mTOR, Erk1/2-MAPK, and Wnt/β-Catenin. In this review, we will focus on these signaling pathways and the crosstalk between them. Given the many factors involved in the process, extending our knowledge in this field will be crucial to understand the hidden mechanisms driving neurodegenerative myelin-associated pathologies.

### 2.1. PI3K/Akt/mTOR

**Figure 1:**
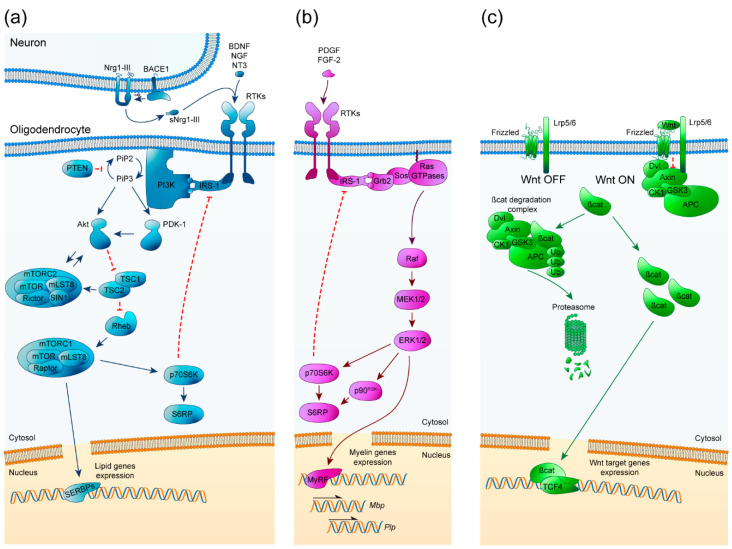
Schematic representation of PI3K/Akt/mTOR, ERK1/2-MAPK, and Wnt/β-catenin pathways. (**a**) PI3K/Akt/mTOR signaling pathway key elements for oligodendrocyte maturation and myelination. Arrowheads imply positive interactions while bars indicate inhibitory signals. BDNF: brain-derived neurotrophic factor, NGF: nerve growth factor, NT3: neurotrophin 3, (s)Nrg1-III: (soluble) Neuregulin 1 type III, BACE1: β-site amyloid precursor protein cleaving enzyme 1, RTKs: receptors tyrosine kinase, IRS-1: insulin receptor substrate 1, PI3K: phosphoinositide-3 kinase, PIP2: phosphatidylinositol (4,5)-biphosphate, PIP3: phosphatidylinositol (3,4,5)-triphosphate, PTEN: phosphatase and tensin homolog, PDK-1: 3-phosphoinositide-dependent protein kinase 1, Akt: protein kinase B, TSC1/2: tuberous sclerosis complex, Rheb: Ras homolog enriched in brain, mTOR: mammalian target of rapamycin, mLST8: target of rapamycin complex subunit LST8, Raptor: regulatory associated protein of mTOR, Rictor: rapamycin-insensitive companion of mTOR, SIN1: Target of rapamycin complex 2 subunit MAPKAP1, p70S6K: Ribosomal protein S6 kinase beta-1, S6RP: Ribosomal protein S6, SREBPs: Sterol regulatory element-binding proteins. (**b**) ERK1/2-MAPK signaling pathway crucial elements for oligodendrocyte maturation and myelination. Arrowheads imply positive interactions while bars indicate inhibitory signals. PDGF: platelet-derived growth factor, FGF-2: fibroblast growth factor 2, RTKs: receptors tyrosine kinase, IRS-1: insulin receptor substrate 1, Grb2: growth factor receptor-bound protein 2, Sos: son of sevenless, MEK1/2: mitogen-activated protein kinase, ERK1/2: extracellular signal-regulated kinases 1 and 2, p70S6K: Ribosomal protein S6 kinase beta-1, p90^RSK^: p90 ribosomal S6 kinase, S6RP: Ribosomal protein S6, MyRF: Myelin regulatory factor. (**c**) Wnt/β-catenin signaling pathway essential elements for oligodendrocyte maturation and myelination. Arrowheads imply positive interactions. Lrp5/6: Lipoprotein receptor-related proteins 5/6, Dvl: disheveled, GSK3: glycogen synthase kinase 3, CK1: casein kinase 1, APC: adenomatous polyposis coli, βcat: β-catenin, TCF4 (TCF2l7): transcription factor 4.

The phosphatidyl inositol-3-phosphate kinase (PI3K)/Akt/mTOR pathway has been studied from a neurodegenerative point of view due to its important role in the regulation of OPC differentiation, myelination, and remyelination [35,36]. It is an ubiquitously expressed and important signaling pathway that is involved in many cellular processes such as cell growth, proliferation, and survival. The PI3K/Akt/mTOR pathway is initiated by the extracellular binding of growth factors like insulin growth factor-1 (IGF-1) to receptor tyrosine kinases (RTKs) at the cell surface, activating them and leading to the initiation of an intracellular cascade. Then, PI3K phosphorylates phosphatidylinositol (4,5)-biphosphate (PIP2) to generate phosphatidylinositol (3,4,5)-triphosphate (PIP3). Consequently, Akt and 3-phosphoinositide-dependent kinase 1 (PDK1) are recruited to the membrane, where Akt is phosphorylated at Thr308 and partially activated. The complete activation of Akt is made by the mechanistic target of rapamycin complex (mTORC2) through its phosphorylation at Ser473. Fully activated Akt then inhibits tuberous sclerosis complex 2 (TSC2) by phosphorylation, a GTPase-activating protein (GAP) that in normal conditions inhibits Rheb. By inhibiting TSC2, Rheb becomes active and allows mTORC1 activation. In addition, the TSC complex, which is formed by TSC1 and TSC2, can activate mTORC2 in an independent Rheb manner [36]. mTORC1 regulates p70S6k and sterol regulatory element-binding proteins (SREBPs) such as SREBP1c and SREBP2, essential transcription factors for the expression of myelin proteins and enzymes involved in lipid synthesis [37] (Figure 1a).

Many in vitro and in vivo studies have shown the relevance of PI3K/Akt/mTOR as a core pathway involved in myelination. Neuregulin-1 (Nrg1), a growth factor from the large family of neuregulins [38], is an important upstream activator of Akt. In the CNS, Nrg1 type III (Nrg1-III) modulates myelin sheath thickness upon its cleavage by β-site amyloid precursor protein cleaving enzyme 1 (Bace-1). Bace-1 knockout mice showed CNS hypomyelination with thinner myelin sheaths and reduced levels of phosphorylated Akt (p-Akt), but no differences in the total number of OPCs or mature OLs were found [39]. However, the role of Nrg1 in CNS myelination remains controversial: Nrg1 improves proliferation, survival, and differentiation of OPCs in vitro through ErbB receptors and can promote remyelination in vivo, but its absence has little to no effect on CNS myelination [40,41].

Initially, the role of Akt in myelination was addressed using mouse models with constitutively activate Akt (Akt-DD). These mice showed significant hypermyelination throughout the CNS, associated with a continuous increase in myelin thickness and myelin proteins, which became pathological as mice aged [42]. Interestingly, no differences were found between the number of OPCs and mature OLs. In this sense, conditional knockouts of phosphatase and tension homolog (PTEN) were generated using Cre recombinase technology under oligodendrocyte transcription factor 2 (Olig2), 2′,3′-Cyclic-nucleotide 3′-phosphodiesterase (CNP) or myelin proteolipid protein (PLP) (Olig2^Cre/+^, CNP^Cre/+^ or PLP^CreERT2/+^ respectively) promoters. The deletion of PTEN, an upstream inhibitor of Akt, achieved a phenotype similar to Akt-DD, with hypermyelination in the CNS due to an increase in PIP3 levels and p-Akt without changes in the total number of mature myelinating OLs [43]. However, inhibition of PTEN was not a successful approach to treat demyelinating diseases, as it does not enhance remyelination following white matter injury [44].

mTOR is recognized as a clear downstream effector of Akt, evidenced by numerous studies. Using rapamycin, Narayanan and colleagues chronically inhibited mTOR in hypermyelinating Akt-DD mice. Concurrent with a decrease in the degree of phosphorylation of downstream effectors (like p70S6K) and expression of myelin proteins, the phenotype was reduced to approximately wild-type levels of myelin production. Accordingly, when rapamycin was administered to young wild-type mice, the rodents exhibited hypomyelination and lower levels of myelin proteins [45]. Several experiments were conducted to describe whether the impact of mTOR on myelination was driven by mTORC1 or mTORC2, depending on mTOR binding to Raptor or Rictor, respectively. Conditional knockout of Raptor induced hypomyelination in the corpus callosum, with thinner myelin sheaths, reduced p-p70S6K and lowered myelin protein levels consistent with a delay in the maturation process, but not a total loss of OLs. However, in the spinal cord, the hypomyelination was more severe and followed a dramatic loss of OLs at P60 [46]. This could be explained by differences in the OL environment or intrinsic OL heterogeneity, as emerging studies have shown the existence of distinct, spatially segregated OL populations [12]. When Raptor ablation was induced in mature OLs, they were unable to maintain proper myelin sheaths, suggesting a role of mTORC1 in the maintenance of adult myelin sheaths [47]. This study also revealed that mTORC1 regulates lipid biosynthesis via SREBPs, as its removal produced a dysregulation of lipid composition [47]. On the other hand, Rictor ablation had mild effects on myelination, as the mice only displayed a partial loss of myelin mRNAs and protein without changes in myelin thickness or proportion of myelinated axons [46]. Interestingly, a lack of Rictor resulted in an increase of mature OLs and a reduction of OPCs during myelination, suggesting a role for mTORC2 in the transition from OPC to OL [47]. Upstream of mTOR, the deletion of TSC during development resulted in hypomyelination [47]. However, there may be different roles for TSC, as its inhibition in OPCs stimulated remyelination but its deletion in OLs slowed remyelination following injury [48]. In addition, aberrant activation of mTORC1 by loss of Tsc1 can affect the correct paranodal domain formation [49]. Another upstream regulator of mTORC1 is Rheb1, which has been implicated in the differentiation from OPCs to mature OLs, but it is not needed for the production or maintenance of new myelin in the adult brain [50]. Recent advances are shedding light on how mTOR promotes myelination: by regulating cytoskeleton dynamics, mTOR induces cellular process expansion and contributes to the expression and correct localization of myelin basic protein (MBP) [51] (Table 1).

To summarize, PI3K/Akt/mTOR signaling is a key pathway involved in CNS myelination through many steps of the process. Akt principal effector, mTOR, is responsible for the initiation of OL differentiation and maturation. mTORC1 plays a critical role in regulating correct myelination through protein and lipid synthesis, while mTORC2 signaling is minor but important. In addition, mTORC1 is needed to maintain healthy myelin sheaths in the adult, even though it does not regulate myelin sheath numbers. Therapeutic strategies focused on mTOR potentiation could be a good approach to enhance remyelination in myelin disorders.

### 2.2. Erk1/2-MAPK

The mitogen-activated protein kinase (MAPK) pathway of extracellular signal-related kinases 1 and 2 (Erk1 and Erk2) has been described as a principal pathway that regulates oligodendroglial development, proliferation, survival, differentiation, and myelination [36,52]. This ubiquitous signaling pathway is involved in many cellular processes, including differentiation, proliferation, and survival. The Erk1/2-MAPK pathway is initiated by activation of RTKs at the cell surface, resulting from the binding of different extracellular growth factors like platelet-derived growth factor (PDGF), fibroblast growth factor-2 (FGF-2) and neurotrophins (NGF, NT3, and BDNF). This is followed by the activation of the Ras GTPase family, which phosphorylates Raf protein (MAP3K), which in turn phosphorylates the mitogen extracellular signal kinases 1 and 2 (MEK1 and MEK2). MEK1 and 2 phosphorylate Erk1 and Erk2 which are translocated to the nucleus; once there, they can regulate expression of the master transcriptional regulator of critical myelin genes MyRF, which control *Mbp* and *Plp* gene transcription [53,54] (Figure 1b).

Erk1/2-MAPK involvement in myelination has been investigated principally in classical knockout mice and mice with sustained activation of Erk1/2. Mice lacking Erk1/2 showed significant hypomyelination and a decrease in myelin mRNAs associated with a persistent reduced protein expression throughout adulthood. The absence of Erk1/2 did not alter the number of mature OLs expressing PLP, but transcript mRNAs of *Mbp* and *Plp* were decreased, leading to a decrease in myelin thickness [53,55]. Conversely, sustained activation of the pathway showed a significant increase in myelin sheath thickness and expression of Myelin-associated glycoprotein (MAG) and MBP in myelinating co-cultures of constitutively active MEK (MEK1DD) expressed in CNP+ OLs [56,57]. This pathway has also been studied by the conditional ablation of upstream regulators of this pathway (FGF Receptor-2 [FGFR2] or tropomyosin receptor kinase B [TrkB]), which resulted in reduced myelin thickness, whereas OPC differentiation and myelination initiation were unaffected [58,59,60]. All these data demonstrate that Erk1/2-MAPK is not necessary for axonal contact, while it is determinant in the correct establishment of myelin thickness according to axon diameter [36]. Another approach made by Ishii and colleagues [61] was using mouse lines in which Erk1/2 activation was upregulated conditionally in a graded manner. They demonstrated that fine-tuning of Erk1/2 signaling strength is critically important for normal OL cell function. Moderate doses of activity led to the reactivation of quiescent mature OLs, increasing myelin thickness and myelin mRNA levels. However, high doses of Erk resulted in late-onset demyelination and axonal degeneration [61]. The importance of balancing this pathway was later confirmed by a study of Suo [62], in which pharmacological inhibition of MEK promoted myelin regeneration after injury in a cuprizone-induced demyelination model (Table 2).

To recap, Erk1/2-MAPK role in CNS myelination is to regulate myelin sheath thickness to match the diameter of the ensheathed axon and its maintenance during adulthood, but it does not influence the initial contact. In remyelinated lesions, it is common to see thinner myelin sheaths around the axons, so pharmacological modulation of this pathway could be a potential strategy to enhance remyelination.

### 2.3. Wnt/β-Catenin

The wingless and integration site (Wnt) intracellular β-catenin signaling cascade has been described as an essential negative regulator of OPC differentiation, myelination, and remyelination [63]. It is an ubiquitous and important signaling pathway that is involved in many cellular processes such as development, growth, metabolism, and stem cell maintenance. The canonical Wnt/β-catenin pathway is initiated with the extracellular binding of Wnt ligands to Frizzled receptors and their co-receptors low-density lipoprotein receptor-related protein 5 and 6 (LRP5/6), at the cell surface. When the pathway is repressed, β-catenin undergoes proteasomal degradation via the β-catenin destruction complex, formed by adenomatous polyposis coli (APC), Axin, glycogen synthase kinase 3B (GSK-3Β) and casein kinase 1 (CK1). When the pathway is activated, the β-catenin degradation complex becomes inactive by binding to LRP5/6, allowing cytosolic β-catenin to accumulate and translocate to the nucleus, where it interacts with intranuclear T-cell factors/lymphoid enhancer factors (TCF/LEF) to activate gene expression [36] (Figure 1c).

Early studies on the role of the Wnt/β-catenin canonical pathway in myelination were performed after the expression of a dominant-active form of β-catenin (DA-Cat) in the OL lineage [64,65]. Olig2^Cre/+^; DA-Cat and CNP^Cre/+^; DA-Cat mice showed a significant decrease in PLP^+^ mature OLs, lower levels of myelin proteins, and/or a decrease in myelin thickness associated with a hypomyelinating phenotype. However, the number of OPCs was normal, and the phenotype became normal as the mice aged, suggesting a delay in myelination caused by inhibition of OPC differentiation via Wnt signaling [64,65]. In vitro studies have tried to clarify the inhibitory effects of the pathway: addition of Wnt3a (an extracellular agonist of Wnt) into cultures [66,67] led to an increase in OPC numbers and a decrease in PLP^+^ (or GalC^+^) mature OLs. Moreover, pharmacological inhibition of GSK-3Β seemed to override Wnt3a effects and promoted OL differentiation, suggesting additional roles for the pathway apart from inhibiting early OPC differentiation [66]. However, additional work evidenced that the Wnt/β-catenin pathway not only has inhibitory effects on OL maturation but is also required for proper myelination. In vitro studies showed that treatment with Wnt molecules increased OPC proliferation and differentiation for proper myelination [68,69,70]. Deletion of APC in mice, an inhibitor of Wnt signaling, caused a decrease in PLP^+^/CC1^+^ mature OLs and a significant decrease of *Plp, Cnp,* and *Mbp* transcripts. Loss of APC resulted in sustained inhibition of differentiation, triggering persistent hypomyelination throughout adulthood, independent of β-catenin signaling [71]. In addition, β-catenin upregulation by Axin2 inhibition had inhibitory effects on myelination, provoking a decrease in the number of mature OLs [72]. On the contrary, Axin upregulation had beneficial effects on remyelination after injury [72].

Downstream of the pathway, β-catenin associated with TCF/LEF factors to promote gene expression. The TEC/LEF family is comprised of four members in mammals, but Tcf7l2 (also known as Tcf4) is the most highly expressed in OLs [64,71,73,74,75,76]. However, the exact role of Tcf4 has been disputed by contrasting studies: while Fancy et al. correlated Tcf4 expression with immature OPCs [64], Fu et al. described a hypomyelinating phenotype in Tcf4^−/−^ mice [73]. Despite these controversial results, it was proposed that the balance between Tcf4 and β-catenin must be responsible for proper myelination [36,74,77]. Furthermore, the Zhao group has defined the stage-dependent functions of Tcf4 to regulate OL lineage development [78]. These stage-dependent functions are mediated through switching binding partners and provide a molecular framework for understanding the context-specific control of CNS myelination [78] (Table 3).

We can conclude that although Wnt/β-catenin canonical signaling is key to maintain OPC populations and inhibit their differentiation into mature OLs, numerous factors from the pathway have their own signaling effects. Members of the β-catenin destruction complex such as APC or Axin can promote OL maturation and stimulate remyelination in specific contexts [71,72], so they are emerging candidates to study new treatments for neurodegenerative diseases derived from a loss of myelin.

### 2.4. Crosstalk Signaling

**Figure 2:**
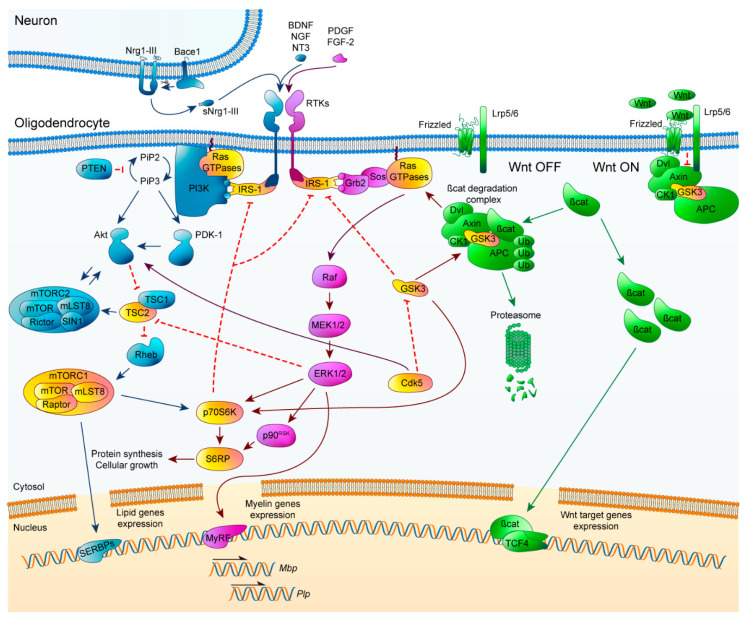
Schematic representation of PI3K/Akt/mTOR, ERK1/2-MAPK, and Wnt/β-catenin pathways and their crosstalk elements. PI3K/Akt/mTOR (blue, left), ERK1/2-MAPK (magenta, middle) and Wnt/β-catenin (green, right). Crosstalk elements between two or more pathways are highlighted in yellow. Arrowheads imply positive interactions while bars indicate inhibitory signals. CDK5: cyclin-dependent kinase 5.

Correct orchestration of PI3K/Akt/mTOR, Erk1/2-MAPK, and Wnt/β-catenin pathways is essential for myelination processes, so learning about how each of the signaling pathways functions by itself is not enough. The highly dynamic environment surrounding OLs means that they receive constant, sometimes conflicting signals which can decide their fate. Every extracellular signal must be assimilated in the specific context of each OL, so the existence of crosstalk elements between pathways is imperative (Figure 2).

First of all, the crosstalk between the PI3K/Akt/mTOR and Erk1/2-MAPK signaling pathways has been approached from different points of view. Both pathways exhibit similar phenotypes after their sustained activation, with an increase in myelin thickness without affecting OL proliferation or differentiation [36,42,43,44,56,57,79]. Inhibition of the PI3K/Akt/mTOR pathway triggers an increase in Erk1/2-MAPK activation, but there is no equivalent reciprocal effect after the inactivation of Erk1/2-MAPK [80]. One of the first elements proposed for this crosstalk was IRS-1, which can activate Erk1/2-MAPK and it is inhibited by mTOR [80]. Another element described for its interaction between these pathways is TSC2. It has been described in tuberous sclerosis, that Erk1/2-MAPK and PI3K/Akt/mTOR can independently phosphorylate and inactivate TSC2 at distinct residues, resulting in activation of mTORC1. Consequently, TSC2 is considered a common element that can regulate mTOR activation, and control of myelination in OLs [81]. Moreover, PI3K/Akt/mTOR downstream element p70S6K was described by Michel et al. for convergence between both pathways within myelination [82]. The use of Erk2 knockout OL-specific mice revealed a decrease in p70S6K activation and S6RP (its downstream target), both of which are fundamental for the transition from premyelinating OLs to mature OLs. This decrease triggered a delay in *Mbp* translation [36,82]. Interestingly, in FGFR2 conditional ablation mice, an attenuation of myelin thickness was observed, accompanied by a significant downregulation of p-mTOR, p-Raptor, and p-S6RP, without changes in p-Akt levels. This revealed that FGFR2 regulates myelin thickness through Erk1/2-MAPK activation and promotes mTORC1 activity in an Akt-independent way, highlighting the importance of mTORC1 as a crosstalk element [83]. Several studies have established that the PI3K/Akt/mTOR and Erk1/2-MAPK intracellular signaling pathways work independently, sequentially, and converge during oligodendroglial lineage progress and myelination. Specifically, Ishii et al. have extensively studied how these two pathways interact [35]. Briefly, they used a series of modified mice to explain how the deficit of different elements of one signaling pathway affected those in the other pathway. Sustained Akt overactivation in OLs of Erk1/2 knockout mice was demonstrated to partially offset the deficit in myelin, p70S6K, and pS6RP expression, but completely rescued p-mTOR expression. Constitutive activation of PI3K in OLs of Erk1/2 knockout mice fully abrogated myelin gene expression deficits, myelin growth, and p-mTOR, pS6RP, and p70S6K. Early loss of mTOR resulted in altered OL differentiation and hypomyelination, which could not be rescued by overexpression of Mek1. However, Mek1 overexpression did reactivate myelin gene expression in adult OLs. In summary, they described that during development and initiation of myelination, PI3K/Akt/mTOR is the key regulator, whereas in active myelination, both pathways are involved and converge at mTORC1. Conversely, Erk1/2-MAPK is the primary pathway during adulthood responsible for the preservation of myelinated axon integrity, with reduced mTOR involvement [35].

Crosstalk between PI3K/Akt/mTOR and Wnt/β-catenin was initially described by rapamycin inhibition of mTOR in in vitro assays, which revealed a significant increase of *Tcf4*, a downstream effector of the Wnt/β-catenin pathway. These results suggest that mTOR allows OL differentiation, altering Wnt canonical pathway signaling [84]. Later, more evidence linked PI3K/Akt/mTOR and GSK-3Β through cyclin-dependent kinase 5 (CDK5), one of a family of serine/threonine kinases that are mainly implicated in regulatory processes of the cell cycle. CDK5 is an atypical member involved in the regulation of neuron differentiation and OL development [85]. When CDK5 was conditionally knocked out in mice, p-Akt significantly decreased while GSK-3Β activity was increased, accompanied by diminished OPC differentiation and myelination following LPC-induced demyelination [36,86]. CDK5′s importance has also been noted for its critical contribution to the architecture of nodes of Ranvier [87].

The crosstalk interactions that directly connect the Erk1/2-MAPK and Wnt/β-catenin pathways during OL development and CNS myelination have yet to be described. Several studies in other cell types have shown that kinase GSK-3Β plays a pivotal role, interacting with multiple signaling pathways. In cancer, it is known that GSK-3Β is phosphorylated and inactivated by Erk1/2-MAPK activation, provoking an upregulation of β-catenin [88,89]. Even though Wnt signaling has been associated with a delay in differentiation, recent studies show that there may be crucial moments during OL development in which Wnt is essential for correct myelination.

Therefore Erk1/2-MAPK may be a part of the complex regulatory mechanisms responsible for tight control of Wnt/β-catenin signaling within OLs. Many non-neuronal cell cultures have pointed to increases in mTOR and p70S6K activity by GSK-3Β [36,90]. PI3K/Akt/mTOR interaction with GSK-3Β takes place at the level of Akt, which can phosphorylate and inactivate it. GSK-3Β interacts with the PI3K/Akt/mTOR pathway to regulate cell proliferation by phosphorylation of p70S6K [91,92]. Even though a direct interaction in signaling has not yet been shown in a myelination context, it is not unreasonable to think that a link between all three pathways could occur in the same way. It is necessary, in the future, to answer this question to clarify the contribution of GSK-3Β between all the pathways.

In summary, IRS-1, TSC2, p70S6K, mTORC1, CDK5, and GSK-3Β are the principal crosstalk elements that have been investigated because alterations to their functionality can completely modify myelination. In this context, there is an element that may be orchestrating all these pathways together and could be upregulated to potentiate remyelination: Ras GTPases, which are upstream initiators of both PI3K/Akt/mTOR and Erk1/2-MAPK pathways, and can coordinate Wnt/β-catenin route through downstream effectors.

### 2.5. The Ras Family of GTPases and Their Role in Neurodegenerative Myelin Diseases

The Ras Superfamily of small GTPases are membrane-anchored intracellular signal transducers that share a conserved structure and biochemical properties. They are known for having low molecular weights (20–30 kDa), and they act as molecular switches that switch signaling pathways on and off by binding and hydrolyzing GTP, respectively [93,94]. In this way, they can transduce extracellular signals to intracellular signaling to regulate proliferation, differentiation, and survival. The Ras superfamily is made up of 150 members that are clustered in 5 families according to their sequence homology: Ras, Rho, Rab, Ran, and Arf [95].

**Figure 3:**
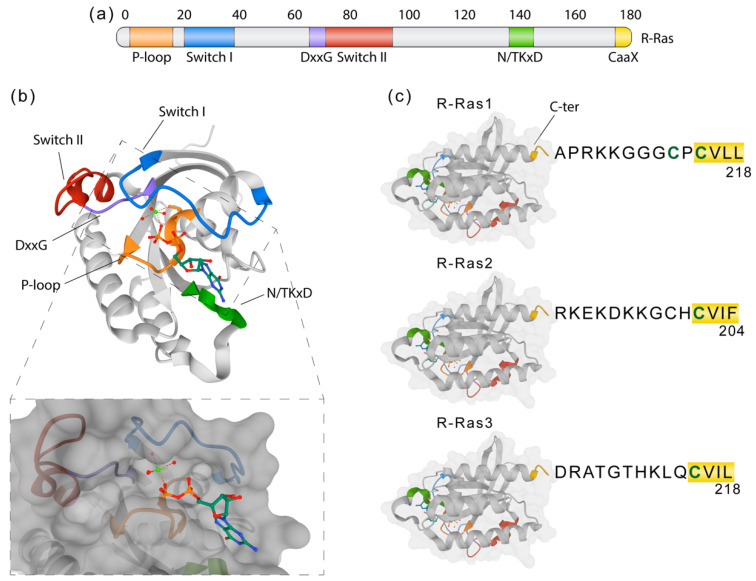
R-Ras molecular structure and domains. (**a**) Representation of the R-Ras primary structure showing domains related to its function. Switch I and II (blue and red) are critical interfaces for downstream effectors and part of the nucleotide-binding pocket. The phosphate-binding loop (P-loop, orange) and the N/TKxD (green) regions are important for binding nucleotides, while the DxxG (violet) motif confers specificity for guanosine nucleotides. The CaaX C-terminal region is crucial for membrane attachment via prenylation or fatty acid modification. (**b**) Crystal structure of R-Ras1 bound to GDP (PDB: 2FN4) with highlighted regions corresponding to the ones in (**a**). GDP is shown as a ball and stick model, with each atom colored by element. The lower box represents the molecular surface model showing how GDP accommodates within the hydrophobic region. (**c**) The C-terminal ends of R-Ras1, R-Ras2, and R-Ras3, which constitute the “hypervariable region” (HVR), show significant sequence diversity important for their subcellular localization. CaaX box is underlined in yellow, with “C” being a cysteine substrate for prenylation, “a” any aliphatic amino acid, and “X” any amino acid.

Members of the classic Ras family (Hras, Kras, and Nras) are frequently mutated and constitutively active in human cancers [96,97]. GTPases of the Ras-related (R-Ras) subfamily, composed of R-RAS1 (RRas), R-RAS2 (TC21), and R-RAS3 (also called MRas), are less well understood [98,99,100]. R-Ras proteins share strong homology (55–60% amino acid identity, Figure 3) with the classic Ras proteins [99,101,102] and with other effector proteins, but many of its functional implications remain undescribed. R-Ras1 and R-Ras2 are ubiquitous [103,104], whereas R-Ras3 expression is more restricted [105,106]. *R-Ras1* encodes a 218-amino acid protein (23.5 kDa) that shares 55% homology with H-Ras, 65% with R-Ras2 and 46% with R-Ras3. R-Ras1 has been described to be expressed in OLs in vitro with a possible implication in maturation from OPCs to myelinating OLs [107]. *R-Ras2* encodes a 204-amino acid protein (23.4 kDa). Some effectors known to be activated by R-Ras1 and R-Ras2 are c-Raf and PI3K [108,109,110,111,112,113].

Recently, R-Ras1 and R-Ras2 have been described to play an essential role in OL differentiation and survival and their absence reduces activation of the PI3K/Akt/mTOR and Erk1/2-MAPK pathways. R-Ras1 and R-Ras2 can signal through PI3K/Akt/mTOR and Erk1/2-MAPK pathways, as their absence produced a significant decrease of p-Thr308-Akt, p-Ser473-Akt, p-Erk1/2, and p-S6RP. Mice lacking R-Ras1 and/or R-Ras2 showed a decrease in mature OL populations and a higher proportion of immature OLs, correlated to an increased expression of Tcf4. Lack of R-Ras1 reduced myelin sheath thickness, while the deletion of R-Ras2 decreased the total number of myelinated axons, with a more severe phenotype in the *R-Ras1^−/−^; R-Ras2^−/−^* mice. This study revealed that R-Ras1 and R-Ras2 play essential and non-redundant functions in correct myelination processes [114], and their absence modifies the major pathways involved in myelination. These data suggest that R-Ras1 and R-Ras2 GTPases are candidate players in crosstalk activities within these important signaling pathways.

## 3. Conclusions

Myelin is essential for the proper function of the nervous system since it facilitates the correct integration of the sensory, motor, and cognitive information. In this sense, the main objective for control of neurodegenerative diseases is the rescue of myelin sheath integrity. In this review, we have illustrated that even though signaling pathways individually contribute to myelination, there is also a complex network of crosstalk elements that thoroughly connects these pathways, thereby controlling the final effects on myelination. In addition, we have highlighted R-Ras1 and R-Ras2 proteins as essential crosstalk elements for the proper coordination and control of myelination processes. A deeper understanding of the molecular mechanisms which coordinate myelination and remyelination processes is the key to establish new promising treatments to fight neurodegenerative myelin-associated diseases.

## Figures and Tables

**Table 1:** Studies supporting the relevance of the PI3K/Akt/mTOR signaling pathway in OL development.

Experimental Approach	Myelin Thickness	Myelination Degree	Phosphorylation of Downstream Elements	Myelin Proteins Expression	Nº of Mature OLs	Nº of OPCs	OL Differentiation	References
*Bace1^−/−^*	-	Normal	-	-	Normal	Normal	Normal	[39]
*Akt-DD ^1^*	+	+	+	+	Normal	Normal	Enhanced	[42]
Rapamycin ^2^	-	-	-	-				[45]
*Pten^fl/fl^; Cnp1^Cre/+^* *Pten^fl/fl^; Plp1^CreERT2/+^*	+	+			Normal	Normal		[43]
*Pten^fl/fl^; Olig2^Cre/+^*	+	+	+		Normal	Normal	Normal	[44]
*CNP^Cre/+^; Raptor^fl/fl^*	-	-	-	-	-	+	Delayed	[46]
*CNP^Cre/+^; Rictor^fl/fl^*	Normal	Slightly -		-	Normal	-	Increased	[46]
*CNP^Cre/+^; Rptor^fl/fl^*	-	-	-	-	Normal	Normal		[47]
*CNP^Cre/+^; Rictor^fl/fl^*	-	Delayed myelination	Normal	Normal	Normal	Normal		[47]
*CNP^Cre/+^; Rptor^fl/fl^; Rictor^fl/fl^*	-	-	-	-	-	+	Delayed	[47]
*CNP^Cre/+^; Tsc1^fl/fl^*		-	+	-				[47]
*Olig1^Cre/+^; Rheb1^fl/fl^*	Normal	-	-	-	-	+	Impaired	[50]
*NG2^Cre/+^; Tsc1^fl/fl^*	+ (later is normal)	Enhanced remyelination		+	Normal	Normal	Normal	[48]
*Plp1^Cre/+^; Tsc1^fl/fl^*	- (later is normal)	Delayed remyelination			Normal	Normal		[48]

(-) indicates lower levels or numbers, (+) indicates higher levels or numbers. ^1^ constitutively active Akt, ^2^ mTOR inhibitor.

**Table 2:** Studies supporting the relevance of the ERK1/2-MAPK signaling pathway in OL development.

Experimental Approach	Myelin Thickness	Myelination Degree/Start of Myelination	Phosphorylation of Downstream Elements	Myelin Proteins Expression	Cellular Proliferation	Nº of Mature OLs	Nº of OPCs	OL Differentiation	References
*Mbp^Cre/+^;* *TrkB^fl/fl^*	-	-/Delayed		-	+	Normal	+	Normal	[60]
*CNP^Cre/+^;* *Fgfr1/2^fl/fl^*	-	-/Delayed	-	-	Normal	Normal	Normal	Normal	[58]
*CNP^Cre/+^;* *Erk1^−/−^; Erk2^fl/fl^*	-	Normal/Normal		-		Normal	Normal	Normal	[53]
*hGFAP^Cre/+^;* *Erk2^fl/fl^* *NG2-^Cre/+^;* *Erk2^fl/fl^*		Slightly -/Delayed			Normal	-	Normal	Delayed	[55]
*CNP^Cre/+^;* *MEK1DD^+/− (1)^*	+	Normal/Enhanced remyelination	+	+	Normal	Normal	Normal	Normal	[53]
*CNP^Cre/+^;* *MEK1DD^+/− (1)^* *Olig1^Cre/+^;* *MEK1DD^+/− (1)^*	+	+/Normal	+	+		Normal	+		[57]
*Olig1^Cre/+^;* *Erk1^−/−^;Erk2^fl/fl^*					-	-	-		[57]

(-) indicates lower levels or numbers, (+) indicates higher levels or numbers. ^(1)^ constitutively active MEK1.

**Table 3:** Studies supporting the relevance of the Wnt/β-catenin signaling pathway in OL development.

Experimental Approach	Myelination Degree	Myelin Proteins Expression	Cellular Proliferation	Nº of Mature OLs	Nº of Immature OLs (OPCs)	OL Differentiation	References
*Olig2^CreERT2/+^; APC^fl/fl^*	-	-	-	-	-	Impaired	[71]
*Axin2^−/−^*	Delayed remyelination		Normal	-	Normal	Delayed	[72]
Treatment with XAV939 ^1^	+	+	Normal	+	-	Enhanced	[72]
GSK-3Β inhibitors	+	+	+	+	+	Normal	[66]
*Olig2^Cre/+^; DA-Cat ^2^*	-	PLP+ decreased		-	Normal	Delayed	[64]
*Cnp^Cre/+^; DA-Cat*	-	-	Normal	-	Normal	Delayed	[65]
*Tcf4^−/−^*	-	-		-		Delayed	[73,74]

(-) indicates lower levels or numbers, (+) indicates higher levels or numbers. ^1^ XAV939 stabilizes Axin2, ^2^ constitutively active β-Catenin.
